# Justice Sensitivity in Middle Childhood: A Replication and Extension of Findings

**DOI:** 10.3389/fpsyg.2020.610414

**Published:** 2021-01-25

**Authors:** Rebecca Bondü, Maria Kleinfeldt

**Affiliations:** Department of Psychology, Psychologische Hochschule Berlin, Berlin, Germany

**Keywords:** justice sensitivity, measurement invariance, stability, middle childhood, longitudinal

## Abstract

Previous research showed justice sensitivity (JS) – the tendency to perceive and negatively respond to injustice as a victim, observer, or perpetrator – to be reliably and validly measurable in middle childhood, but unexpected findings concerning mean values and measurement invariance (MI) require replication, and retest reliabilities, longitudinal relations with prosocial and aggressive behavior, and relations with teacher ratings are currently unknown. This study, therefore, examined mean values, factor structure, retest reliabilities, and MI of self- and parent-rated JS as well as their relations with parent- and teacher-rated prosocial and aggressive behavior and a range of social skills in a sample of 1,329 children between 5 and 12 years of age (first measurement: *M* = 8.05, SD = 1.02, 51.1% girls). Using self- and parent ratings, we could replicate the intended factor structure of three related yet distinct JS subscales (victim, observer, and perpetrator). We found strong MI between those ratings. Retest reliabilities of parent ratings were similar to older age groups, but lower for self-ratings. All JS perspectives were positively related with theory of mind and empathy, indicating a good understanding of others’ internal states. Victim JS was negatively related to affective and behavioral self-regulation, whereas observer and perpetrator JS showed positive relations. Victim JS negatively and observer and perpetrator JS positively predicted prosocial behavior. The opposite pattern was found regarding aggressive behavior. This study provides additional support that JS can be measured via self- and other reports in childhood and that it may influence behavior early on. It adds to explaining the relations with prosocial and aggressive behavior.

## Introduction

Research showed that differences in the tendency to perceive and adversely respond to injustice – justice sensitivity (JS) – can be measured from middle childhood onward (Strauß et al., 2020). Some of these findings, however, contrasted findings in older age groups or theoretical assumptions underlying JS and require replication. Furthermore, retest reliabilities and longitudinal associations with behavior were not yet examined in children. Finally, associations between JS and social skills were investigated, but the findings were sometimes surprising, only relied on parent reports, and did not include important skills, such as cognitive flexibility. The present study used an independent sample of 1,329 children and three sources of information (children, parents, and teachers) to replicate and extend previous findings and add to the knowledge about JS and its measurement in middle childhood. The replication of unexpected results in previous research is important considering the current replication crisis in psychology. Furthermore, given that trait measurement in children is often challenging, it is important to ensure that JS can be reliably and validly measured before using the measure in further research.

### Justice Sensitivity

Individuals high in JS perceive injustice frequently and negatively respond to injustice, for example by rumination and strain (Schmitt et al., 2005). JS refers to typical instances of injustice (e.g., being treated worse than others) and does not only apply to specific forms of injustice (e.g., moral injustice). JS is a small yet distinct personality trait that is related to, but could be separated from partly overlapping constructs, such as neuroticism and the other Big Five, hostility, trait anger, reactive aggression, empathy, and similar sensitivity traits, such as rejection sensitivity (Schmitt et al., 2005, 2010; Bondü and Richter, 2016). The primary affective response is determined by the perspective from which individuals are sensitive to injustice: victim-justice-sensitive individuals tend to feel unjustly treated, respond by anger, and retaliate (Schmitt et al., 2005). Victim JS reflects an egoistic interest in justice and was consistently related to more aggressive and uncooperative and less pro social behavior (Fetchenhauer and Huang, 2004; Gollwitzer et al., 2005; Strauß et al., 2020). Observer-justice-sensitive individuals tend to perceive injustice inflicted onto others, respond by indignation, and wish for victim compensation or perpetrator punishment. Perpetrator-justice-sensitive individuals tend to perceive themselves as treating others unfairly, respond with guilt, and wish for victim compensation or self-punishment (Schmitt et al., 2010). Observer and perpetrator JS reflect an altruistic interest in justice and were associated with more prosocial and cooperative and less aggressive behavior (Gollwitzer and Rothmund, 2011; Bondü and Elsner, 2015; Bondü, 2018; note that beneficiary JS was not examined in children yet).

All JS perspectives were positively correlated, reflecting a common concern for justice (Schmitt et al., 2010). Correlations are typically largest between observer and perpetrator (0.5–0.6) and smallest between victim and perpetrator JS (about 0.3). When using self-reports, mean values were highest for victim and lowest for perpetrator JS (Schmitt et al., 2010; Bondü and Elsner, 2015; Strauß et al., 2020). The first study that used parent reports found the opposite pattern, pointing to influences of social desirability on self-ratings or to particularities of the previous sample (Strauß et al., 2020). Hence, this finding requires replication.

A factor structure of correlated but separable subscales was found in adolescents and adults, with self- and parent reports and different JS measures. Stability rates ranged between 0.5 and 0.6 for all perspectives (Schmitt et al., 2010; Bondü et al., 2016). Out of two studies in middle childhood, only one could replicate this factor structure (Ehrhardt-Madapathi et al., 2018; Strauß et al., 2020). Furthermore, retest reliabilities were not yet examined in children.

Research established strong measurement invariance (MI) for JS between points of measurement, self- and parent reports, and boys and girls (Schmitt et al., 2010; Bondü et al., 2016). Given the differences in mean-level values, the findings of strict MI between self- and parent ratings in middle childhood were surprising, especially because their correlations were small (Strauß et al., 2020). This finding may be due to *z*-standardization of self- and parent ratings to make them comparable, resulting in the leveling of mean-level differences. Hence, different approaches require consideration when examining MI between self- and parent reports.

Finally, research indicated increases in victim JS and minor changes in observer and perpetrator JS during adolescence (Bondü and Elsner, 2015). Hence, observer and perpetrator JS might already increase in middle childhood.

### Further Research Questions

Research showed no associations between victim JS and prosocial behavior in adolescents and adults (Fetchenhauer and Huang, 2004; Bondü and Elsner, 2015), but negative links were found in middle childhood (Strauß et al., 2020). Prosocial behavior was predicted by observer *or* perpetrator JS in older age groups, presumably due to large amounts of shared variance. In middle childhood, both added to this prediction. Hence, the question arises whether the links between JS and prosocial behavior differ between age groups or whether this finding was specific to the previous sample.

Given its negative relations with prosocial behavior and positive relations with antisocial behavior and anger, victim JS was expected to be negatively related to social skills. However, all JS perspectives showed positive relations with empathy and theory of mind (ToM) in child and adult samples (Schmitt et al., 2005; Baumert et al., 2014a,b; Decety and Yoder, 2016). Again, the question arises whether these findings can be replicated in another sample. Finally, research related JS only to single aspects of parent-rated self-regulation (Strauß et al., 2020). Other aspects of self-regulation, such as emotional control, cognitive flexibility, and working memory, were not investigated and other sources of information were not considered.

### The Present Study

This study examined whether the findings on JS in middle childhood in previous research can be replicated in an independent sample, and also studied retest reliabilities, longitudinal relations between JS and prosocial and aggressive behavior, and cross-sectional associations with a broad range of social skills rated by parents and teachers. We expected to replicate previous research findings even if they contradicted prior assumptions. We expected a replication of the intended factor structure (Hypothesis 1), contrasting patterns of mean-level values in self- and parent ratings (Hypothesis 2), negative relations between victim JS and prosocial behavior (Hypothesis 3), the prediction of prosocial behavior by observer *and* perpetrator JS (Hypothesis 4), and similar correlation patterns with social skills in parent and teacher ratings (Hypothesis 5). We also expected retest reliabilities to be similar to previous research (Hypothesis 6), increases in observer and perpetrator JS with age (Hypothesis 7), and longitudinal associations between JS and prosocial/aggressive behavior (Hypothesis 8). Contrasting previous findings, we expected weak MI between self- and parent ratings (Hypothesis 9) (see table with all the hypotheses and findings in the Supplementary Material).

## Method

### Sample

We collected data for *N* = 1,329 children at first measurement (T1). One thousand three hundred and fifteen 5- to 12-year-olds (*M* = 8.05, SD = 1.02, 51.1% girls), 848 parents, and 1,098 teachers filled in the questionnaires. Of the children, 13.1% attended first, 31.9% second, 28.8% third, and 18.3% fourth grade and 7.9% cross-year learning; 68.7% attended schools in Brandenburgia and 31.1% in Berlin, Germany. At the second measurement (T2), we collected data for 1,177 children (1,154 child, 548 parent, and 617 teacher questionnaires). The average retest interval was 10.96 months (SD = 1.33).

### Measures

We obtained information from children, parents, and teachers. To keep the required time to a minimum, respectively, we collected assessments from the source that we considered best suitable to provide the information.

#### Justice Sensitivity

We measured victim, observer, and perpetrator JS *via* self- and parent ratings with a simplified version of the five-item Justice Sensitivity Inventory for Children and Adolescents (Bondü and Elsner, 2015; Strauß et al., 2020 for exact item wordings). Response options ranged from 0 = *not at all true* to 3 (self-ratings) or 5 (parent ratings) = *exactly true*. Each perspective was measured with five congruently worded items (victim JS: “I cannot easily bear it when others take advantage of me.”; observer JS: “…when someone takes advantage of others.”; perpetrator JS: “I cannot easily bear the feeling of taking advantage of others.”). The same 15 items were reworded for parent ratings (“My child cannot easily bear it when others take advantage of them.”).

#### Prosocial Behavior

We assessed children’s prosocial behavior over the past 6 months *via* parent ratings at T1 and T2 and teacher ratings at T1 with five items from the Strengths and Difficulties Questionnaire (Goodman, 1997; “My/The child is kind to younger children”). Response options ranged from 0 = *not at all* to 2 = *definitely.*

#### Aggressive Behavior

We assessed aggressive behavior in the past 6 months *via* parent ratings at T1 and T2 and *via* teacher ratings at T1 with eight adapted items from the Children’s Social Behavior Scale (Crick, 1996; “Hits or pushes others.”). Response options ranged from 0 = *never* to 4 = *daily*.

#### Empathy

We assessed empathy *via* translated items of the Basic Empathy Scale (Jolliffe and Farrington, 2006). Parents rated cognitive (9 items; “My child finds it hard to know if others are frightened”) and affective empathy (11 items; “My child doesn’t become sad when it sees other people crying”). Teachers rated children’s affective empathy *via* eight items. Response options range from 0 = *not at all* to 3 = *exactly*.

#### Theory of Mind

We assessed ToM over the past 6 months *via* translated and adapted items from the Theory of Mind Inventory (Hutchins et al., 2012). Parents evaluated their child’s cognitive ToM (“My child understands the word ‘think”’) with 10 items, and teachers rated children’s cognitive and affective ToM (“The child understands that the situation is unsafe or dangerous when I show fear”) with 10 items each. Response options ranged from 0 = *not at all* to 4 = *absolutely*.

#### Emotional Control

Parents rated children’s emotional control over the past 6 months by 10 items from the emotional control subscale of the Behavior Rating Inventory of Executive Function (Gioia et al., 2000; e.g., “My child has explosive, angry outbursts”). Response options ranged from 0 = *never* to 4 = *always*.

#### Anger Reactivity

Parents rated children’s anger reactivity over the past 6 months *via* seven items from the anger/frustration subscale of the Temperament in Middle Childhood Questionnaire (Simonds and Rothbart, 2004; e.g., “Gets angry when he/she makes a mistake.”). Response options ranged from 0 = *not true at all* to 4 = *exactly true*.

#### Executive Functions (Inhibition, Working Memory, and Flexibility)

Parents and teachers rated children’s inhibition with six TMCQ items (Simonds and Rothbart, 2004) and two (parents) or three (teachers) BRIEF (Gioia et al., 2000) items (“Can stop him/herself from doing things too quickly”). They assessed working memory with 10 (“Forgets what he/she was doing”) and flexibility with eight items (“Becomes upset by new situations.”) from the BRIEF. Response options ranged from 0 = *not at all true* to 4 = *exactly true*.

### Procedure

Data from children were collected in 45- to 60-min sessions in schools. Student instructors read out instructions, items, and response options, and children marked their responses. Standardized answers to potential questions were provided. Questions and response options were repeated and shown in the questionnaire to ensure children’s comprehension. First graders were tested in groups of 2–3, second graders in groups of 7–10, and third graders with all participating children of the class. Children were asked not to read out or copy answers. They received gifts for participating. Parents and teachers filled in questionnaires on paper or online. Teachers received a 5€ donation per questionnaire for the class. Written informed consent was obtained from all caregivers. Children, parents, and teachers participated voluntarily and were guaranteed privacy. Questionnaires and proceedings were approved of by the school authorities and an ethics committee.

### Analysis

We computed mean scores for all variables separately for subscales and raters. Manifest analyses were computed using SPSS 25. Latent data analyses were done using M*plus* 8 (Muthén and Muthén, 1998-2012). We used cross-sectional T1 data for descriptive and correlation analyses, T2 JS data to compute retest reliabilities, and T2 prosocial and aggressive behavior data in regression analysis with T1 JS as the predictor. We used confirmatory factor analyses to replicate the intended JS factor structure separately for self- and parent reports. In line with previous research (Schmitt et al., 2005; Strauß et al., 2020) and methodological considerations (Little et al., 2002), we used two-item parcels per subscale as indicators for the latent factors (items 1–3 for the first and items 4–5 for the second parcel per perspective) in all models. An indicator factor with loadings of all second parcels accounted for shared variance and similar wordings between JS perspectives. We examined MI between self- and parent ratings using the proportion of maximum scaling (POMS) method (Little, 2013). POMS involves changing the scale minimum to zero and dividing the scores by the highest value. Thus, the proportions of distances between responses and differences in intercepts are maintained. When examining MI, we modeled separate indicator factors for self- and parent ratings and allowed corresponding JS perspectives to correlate between raters. We inspected χ^2^-difference test, absolute fit indices, and decreases in comparative fit index (CFI) values to determine the level of MI. In order to examine associations between JS perspectives and prosocial and aggressive behavior, we conducted latent path analyses controlling for age and gender. Aggression and prosocial behavior were indicated by two parcels each. The full information maximum likelihood procedure provided in M*plus* accounted for missing data. A robust maximum likelihood estimator accounted for non-normally distributed data. The complex command and class as a cluster variable accounted for children’s clustering in classes. Model fits were considered acceptable if absolute fit indices were acceptable.

## Results

### Descriptive Statistics

**FIGURE 1:**
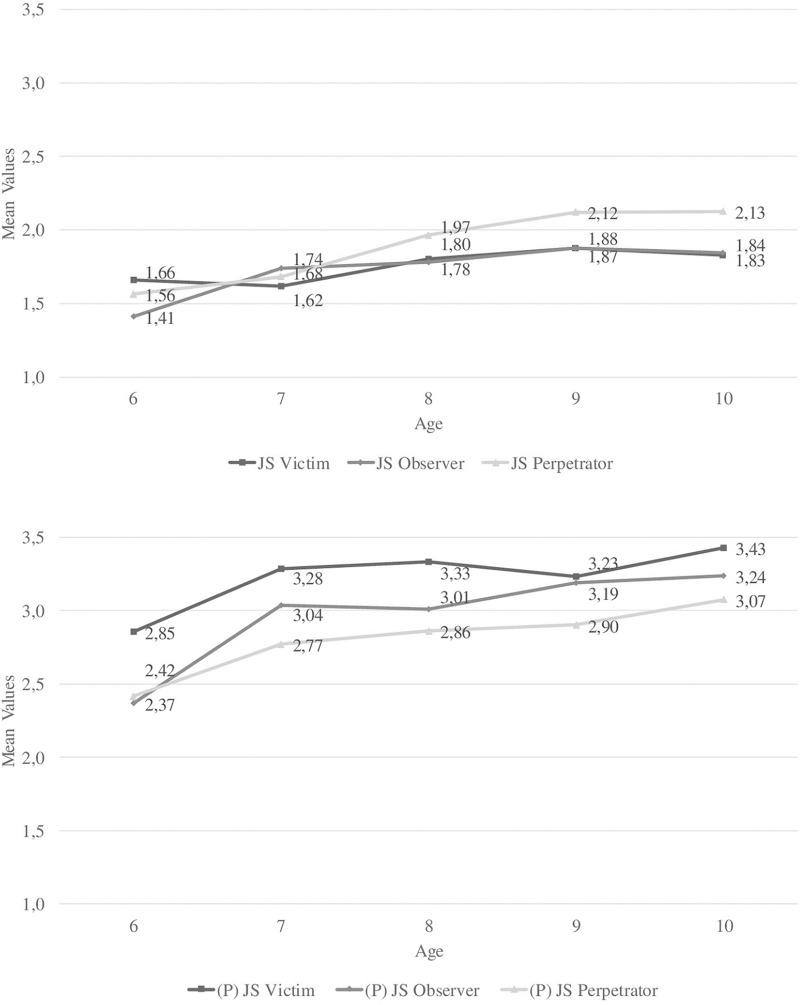
Mean-level differences in self- (range: 0–3) and parent-rated (range: 0–5) justice sensitivity perspectives between age groups.

Table 1 shows ranges, internal consistencies, means, standard deviations, and retest reliabilities for self- and parent-reported JS at T1 and T2. Mostly in line with previous findings, internal consistencies ranged between α = 0.63 and 0.77 for self-reports and between 0.79 and 0.91 for parent reports (lowest: victim, highest: perpetrator, respectively). In line with previous self-report research, children reported the lowest mean values for victim and the highest for perpetrator JS at T1 (no differences between victim and observer JS at T2). Supporting Hypothesis 2, parent ratings showed the opposite pattern of mean-level values of child-ratings at both T1 and T2. Supporting Hypothesis 7, correlations (Table 2) and Jonckheere–Terpstra tests using cross-sectional T1 data indicated significant trends toward higher self-reported victim (*p* < 0.001), observer (*p* < 0.01), and perpetrator JS (*p* < 0.001) as well as parent-reported observer (*p* < 0.01) and perpetrator JS (*p* < 0.05) (Figure 1) with age. Two repeated-measures MANOVAs comparing T1 and T2 ratings indicated a main effect of measurement point for self-reports [*F*(1, 900) = 30.788, *p* < 0.001, η^2^_p_ = 0.033], but not for parent reports. Subsequent analyses revealed significant trends toward higher self-reported victim, observer, and perpetrator JS. Supporting Hypothesis 6, retest reliabilities ranged between *r*_tt_ = 0.510 and 0.593 for parent reports, but only between *r*_tt_ = 0.163 and 0.316 for self-reports.

**TABLE 1:** Descriptive statistics of all variables in the present study.

**Variables**	**Range**	**Self-ratings**				**Parent ratings**				**Teacher ratings**	
		**α**	**T1: *M* (*SD*)**	**T2: *M* (*SD*)**	***r*_tt_**	**α**	**T1: *M* (*SD*)**	**T2: *M* (*SD*)**	***r*_tt_**	**α**	**T1: *M* (*SD*)**
JS victim	0–3/0–5	0.625	1.74 (0.73)	1.87 (0.70)	0.217***	0.788	3.28 (1.02)	3.33 (1.04)	0.593***		
JS observer	0–3/0–5	0.674	1.78 (0.74)	1.85 (0.71)	0.163***	0.903	3.08 (1.12)	3.06 (1.08)	0.510***		
JS perpetrator	0–3/0–5	0.769	1.90 (0.86)	2.03 (0.85)	0.361***	0.907	2.85 (1.23)	2.91 (1.17)	0.558***		
Aggressive behavior	0–4					0.847	0.58 (0.536)	0.62 (0.530)		0.934	0.62 (0.711)
Prosocial behavior	0–2					0.655	1.65 (0.326)	1.65 (0.327)		0.861	1.51 (0.477)
Affective empathy	0–3					0.775	1.95 (0.440)			0.846	1.89 (0.505)
Cognitive empathy	0–3					0.848	2.31 (0.441)				
Affective ToM	0–4									0.889	2.99 (0.610)
Cognitive ToM	0–4					0.887	2.88 (0.646)			0.938	2.83 (0.690)
Emotional control	0–4					0.928	1.21 (0.828)				
Anger reactivity	0–4					0.807	1.67 (0.727)				
Inhibition	0–4					0.696	2.65 (0.605)			0.892	2.77 (0.820)
Working memory	0–4					0.909	1.38 (0.796)			0.957	1.30 (0.988)
Flexibility	0–4					0.799	1.27 (0.557)			0.906	1.14 (0.690)

***p ≤ 0.001.

**TABLE 2:** Correlations of JS perspectives with another and with other variables.

	**2**	**3**	**4**	**5**	**6**	**Prosocial (P)**	**Prosocial (T)**	**Aggr (P)**	**Aggr (T)**	**Cogn Emp (P)**	**Aff Emp (P)**	**Aff Emp (T)**	**Cogn ToM (P)**	**Cogn ToM (T)**	**Aff ToM (T)**	**Emot Contr (P)**	**Anger Reac (P)**	**Inhibition (P)**	**Inhibition (T)**	**WM (P)**	**WM (T)**	**Flexibility (P)**	**Flexibility (T)**	**Age**
1 JS victim T1 (self)	0.471***	0.363***	0.035	0.085*	0.045	0.067	–0.038	–0.027	–0.027	0.135***	0.070	–0.005	0.098**	0.045	0.033	0.029	–0.045	0.092*	–0.009	0.061	0.030	0.065	–0.034	0.127***
2 JS observer T1 (self)		0.492***	0.070*	0.118**	0.076*	0.084*	0.045	–0.068	–0.040	0.117**	0.070	0.063*	0.210***	0.067*	0.084**	0.037	–0.037	0.085*	0.002	0.047	0.029	0.079*	–0.019	0.089**
3 JS perpetrator T1 (self)			0.064	0.163***	0.151***	0.111**	0.054	−0.076*	–0.052	0.175***	0.086*	0.054	0.236***	0.127***	0.152***	0.054	−0.073*	0.180***	0.091**	0.117**	0.143***	0.050	0.085**	0.189***
4 JS victim T1 (parent)				0.380***	0.162***	−0.124***	−0.085*	0.220***	0.088*	0.042	0.170***	–0.035	0.090*	–0.018	–0.010	−0.278**	0.323**	−0.129***	–0.030	−0.073*	–0.009	−0.221***	−0.078*	0.042
5 JS observer T1 (parent)					0.605***	0.305***	0.109**	−0.106**	−0.105**	0.404***	0.356***	0.143***	0.241***	0.061	0.118**	0.020	0.010	0.155***	0.132**	0.109**	0.096*	0.007	0.072	0.114**
6 JS perpetrator T1 (parent)						0.448***	0.211***	−0.319***	−0.212***	0.436***	0.357***	0.206***	0.275***	0.130**	0.173***	0.165**	−0.156**	0.327***	0.229***	0.178***	0.191***	0.109**	0.177***	0.097**

(P), parent report; (T), teacher report; Prosocial, prosocial behavior; Aggr, aggressive behavior; Cogn Emp, cognitive empathy; Aff Emp, affective empathy; Cogn ToM, cognitive theory of mind; Aff ToM, affective theory of mind; Emot Contr, emotional control; Anger Reac, anger reactivity; WM, working memory. *p ≤ 0.05. **p ≤ 0.01. ***p ≤ 0.001.

### Factor Structure and Measurement Invariance

Supporting Hypothesis 1, we replicated the factor structure of three positively correlated yet distinct subscales for self- and parent ratings with two separate CFA first [self-ratings: χ^2^(*df* = 3) = 11.251, *p* = 0.010, CFI = 0.994, root mean square error of approximation (RMSEA) = 0.046 (0.019; 0.076), standardized root mean squared residual (SRMR) = 0.012; *N* = 1,313; parent ratings: χ^2^(*df* = 3) = 3.519, *p* = 0.318, CFI = 1.000, RMSEA = 0.014 (0.000; 0.062), SRMR = 0.010; *N* = 843]. All JS perspectives were positively correlated [highest: observer–perpetrator (*r*_self_ = 0.492, *r*_parent_ = 0.605); lowest: victim–perpetrator (*r*_self_ = 0.363, *r*_parent_ = 0.162)]. Correlations between corresponding self- and parent ratings were small (*r* = 0.035–0.151).

We compared models indicating configural [equivalent models; χ^2^(*df* = 32) = 39.392, *p* = 0.173, CFI = 0.998, RMSEA = 0.013 (0.000; 0.026), SRMR = 0.013], weak [corresponding factor loadings constrained equal; χ^2^(*df* = 39) = 58.524, *p* = 0.023, CFI = 0.995, RMSEA = 0.019 (0.007; 0.029), SRMR = 0.019; Δχ^2^ = 17.736, Δ*df* = 7, Δ*p* = 0.013], strong [corresponding factor loadings and intercepts constrained equal; χ^2^(*df* = 42) = 66.200, *p* = 0.010, CFI = 0.993, RMSEA = 0.021 (0.010; 0.030), SRMR = 0.022; Δχ^2^ = 7.971, Δ*df* = 3, Δ*p* = 0.047], and strict MI [corresponding factor loadings, intercepts, and error variances constrained equal; χ^2^(*df* = 48) = 560.330, *p* < 0.001, CFI = 0.860, RMSEA = 0.090 (0.083; 0.097), SRMR = 0.064; all *N* = 1,320; Δχ^2^ = 475.797, Δ*df* = 6, Δ*p* < 0.001; note that this model showed an error message, but even a model adapted to modification indices did not show better absolute fit indices than the previous model] between self- and parent ratings. Contrasting findings from previous research using *z*-standardization, significant results of χ^2^-difference tests indicated configural MI, but inspections of fit indices indicated strong MI using the POMS approach. Thus, contrasting Hypothesis 9, we found strong rather than weak MI between self- and parent ratings.

### Relations Between JS and Social Behavior and Social Skills

Table 2 shows correlations between self- and parent-reported JS and parent- and teacher-reported social behavior and skills. Supporting Hypotheses 5, parent-rated JS correlated with most variables in the expected direction. Supporting Hypothesis 3, parent-rated victim JS was negatively related with parent-rated prosocial behavior, inhibition, working memory, flexibility, and emotional control. It was positively related with aggression, anger reactivity, affective empathy, and cognitive ToM. Parent-rated observer JS was positively related with parent-rated prosocial behavior, cognitive and affective empathy, cognitive ToM, inhibition, and working memory and negatively with aggression. Parent-rated perpetrator JS was positively related to parent-rated prosocial behavior, empathy, cognitive ToM, emotional control, inhibition, working memory, and flexibility and negatively with aggression and anger reactivity. Also supporting Hypothesis 5, teacher-rated social behavior and skills showed similar but less pronounced correlation patterns (particularly regarding victim JS). Similar correlation patterns emerged for self-rated perpetrator JS and observer JS, but less so for victim JS. Relations of self-rated JS with parent ratings were closer than with teacher ratings.

### Prediction of Prosocial and Aggressive Behavior

**FIGURE 2:**
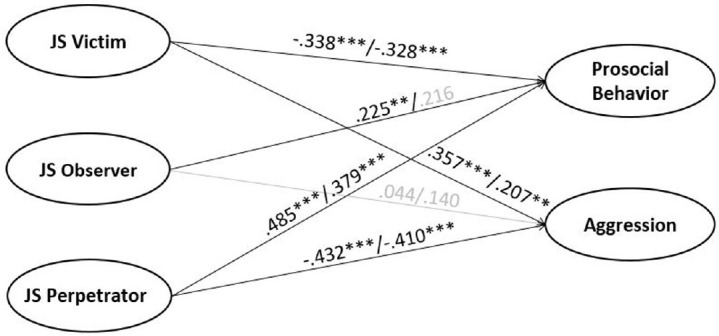
Predicting prosocial behavior and aggression from victim, observer, and perpetrator justice sensitivity (cross-sectional prediction/longitudinal prediction). Controlled for age and gender. Cross-sectional Model: χ^2^(34, *N* = 846) = 119.199, *p* < 0.001, RMSEA = 0.054, CFI = 0.975, SRMR = 0.025; Longitudinal Model: χ^2^(34, *N* = 932) = 86.345, *p* < 0.001, RMSEA = 0.041, CFI = 0.983, SRMR = 0.025.

When predicting parent-rated prosocial and aggressive behavior from parent-rated T1 JS and controlling for age and gender, the model explained 45.7% variance in prosocial and 29.3% in aggressive behavior [χ^2^(*df* = 34) = 119.199, *p* < 0.001, CFI = 0.975, RMSEA = 0.054 (0.044; 0.065), SRMR = 0.025, *N* = 846]. Supporting Hypothesis 4, victim JS predicted less prosocial and more aggressive behavior, perpetrator JS showed the opposite pattern, and observer JS positively predicted prosocial behavior (Figure 2). A similar pattern of findings emerged when using T1 teacher ratings of prosocial and aggressive behavior, but observer JS did not add to these predictions [χ^2^(*df* = 34) = 142.489, *p* < 0.001, CFI = 0.975, RMSEA = 0.054 (0.045; 0.063), SRMR = 0.018; *N* = 1,098]. When predicting parent-rated T2 prosocial and aggressive behavior from parent-rated T1 JS, the model explained 32.4% variance in prosocial and 20.6% in aggressive behavior [χ^2^(*df* = 34) = 86.345, *p* < 0.001, CFI = 0.983, RMSEA = 0.041 (0.030; 0.051), SRMR = 0.025; *N* = 932]. The findings resembled cross-sectional findings (but no significant effect of observer JS remained), supporting Hypothesis 8, but only when the stability of prosocial and aggressive behavior was not considered. When adding T1 prosocial and aggressive behavior, no significant effects remained. When using self-reported T1 JS as a predictor for parent-rated T1 prosocial and aggressive behavior, there were only marginally significant links between victim JS and parent-rated T1 aggressive behavior (β = 0.118, *p* = 0.061) and perpetrator JS and prosocial behavior [β = 0.140, *p* = 0.065; χ^2^(*df* = 34) = 93.542, *p* < 0.001, CFI = 0.978, RMSEA = 0.036 (0.028; 0.045), SRMR = 0.019; *N* = 1,323]. There were no longitudinal associations.

## Discussion

This study aimed to replicate previous findings on JS in middle childhood in an independent sample and to extend the present knowledge on the stability of JS in childhood, relations with teacher ratings of social behavior and skills, and longitudinal relations with prosocial and aggressive behavior. Findings were mainly in line with the hypotheses and previous research. They indicate that JS can be reliably and validly measured from childhood onward and is associated with social behavior early on.

### Descriptive Statistics

Retest reliabilities of parent ratings were similar to older age groups (Schmitt et al., 2005; Bondü and Elsner, 2015), indicating that JS is a stable trait early on. Retest reliabilities of self-reports were lower but indicated that self-perceptions of being someone who tends to feel unjustly treated (victim JS) and/or fears to treat others unfairly (perpetrator JS) start to consolidate in middle childhood. Retest reliabilites were significant but low for self-rated observer JS, suggesting that children struggle to distinguish observer JS from victim and/or perpetrator JS or that disliking injustice inflicted onto others is more open to change in this age range. Supporting previous assumptions, self- and parent reports showed increases in observer and perpetrator JS with age cross-sectionally (only self-reports longitudinally). This indicates an increasing understanding of (in)justice for others in this age range and the comparability of self- and parent reports.

### Factor Structure and Measurement Invariance

Supporting previous research, self-reported JS was highest for perpetrator and lowest for victim JS (Schmitt et al., 2010), whereas parent ratings showed the opposite pattern (Strauß et al., 2020). Thus, high levels of self-rated perpetrator JS may reflect influences of social desirability. Despite this finding and small correlations between self- and parent-reported JS, we replicated the factor structure of positively related yet distinct JS subscales for self- and parent ratings and found strong MI when using the POMS method. Hence, children and parents apparently understand the JS measure and items similarly, but evaluate them differently as evident by the small correlations between their ratings. This may be due to social desirability and/or because children may have difficulties imagining how they would generally react in the presented situations, regardless of whether or not they have actually experienced them before. Parent ratings may be more strongly guided by children’s observable behavior, that is, responses toward perceived injustice rather than insights into children’s cognitions and emotions. This may also be indicated by stronger relations between parent-rated JS with social skills and prosocial and aggressive behavior. Parent ratings may, thus, be more reliable for young children, whereas self-ratings may be more reliable in older children. Nevertheless, ratings can be compared, differences in mean-level orders of the JS subscales are not as influential, and valid and reliable self- and parent ratings can be obtained from middle childhood onward.

### Relations With Social Skills

In line with previous research, all self- and parent-rated JS perspectives showed positive relations with parent- and teacher-rated cognitive and affective ToM and empathy. Hence, children high in JS tend to have an understanding of others’ cognitions and emotions, and positive relations between victim JS and aggressive behavior are not due to a lack of this understanding. Instead, these children tend to show strong anger reactivity and low emotional control (Strauß et al., 2020). Hence, they apparently have difficulties in emotion, but also in behavior regulation: Both parent and teacher reports indicated low executive functions among victim-sensitive children. This may prevent them from capturing all relevant aspects of an ambiguous or unfair situation (working memory), to constrain primary behavioral impulses (inhibition), and to effectively plan and carry out adequate behavioral responses (all EF; Morra et al., 2018). Low cognitive flexibility may furthermore reflect a rigid understanding of justice norms. In contrast, children high in observer and perpetrator JS showed high affective and behavioral self-regulation. Similar patterns of parent and teacher ratings indicate the reliability of findings, even if correlations with teacher ratings tended to be smaller, presumably due to differences in raters of JS.

### Relations With Social Behavior

Also in line with previous research (Gollwitzer et al., 2009; Bondü, 2018; Strauß et al., 2020), victim JS was negatively related to prosocial and positively related to aggressive behavior, whereas perpetrator JS showed the opposite pattern. Observer JS again added to the prediction of prosocial behavior beyond perpetrator JS cross-sectionally. This study extended previous findings to longitudinal data and teacher ratings. Longitudinal relations remained stable only if initial levels of prosocial and aggressive behavior were not controlled (presumably due to stabilities > 0.78). The longitudinal relations may be stronger with a longer lag between both measurements. Future studies may, therefore, examine them across multiple and longer intervals. Self-rated JS was unrelated to parent-rated prosocial and aggressive behavior, indicating that children’s self-perceptions as considering justice as important are unrelated to their social behavior or reflect differences between raters.

### Limitations and Outlook

The strengths of the present study include the sample size, the use of longitudinal data, multiple raters and measures, and its aim to counter the replication crisis in psychological research. Limitations include low internal consistencies of self-rated victim and observer JS [although low internal consistencies are common in child ratings (Measelle et al., 2005; Tackett et al., 2019)] and parent-rated prosocial behavior and inhibition and only relying on questionnaire data. Low internal consistencies of prosocial behavior may be due to little variance because of ceiling effects and because items were rated on a three-point scale. Inhibition is a heterogeneous construct and the items cover variable aspects of inhibition (cognitive and behavioral aspects). Future research should, therefore, consider other sources of information (e.g., experimental/observational data).

Given the evidence for its reliable measurement and early relations with social skills and behavior, JS should receive more attention in psychological research on behavior and well-being in childhood. It appears to influence pro- and antisocial behavior early on. This influence should be considered in future intervention studies by, for example, lowering victim JS through cognitive restructuring and self-regulation training in order to reduce antisocial behavior. Justice-related parenting behavior and education with a focus on other’s needs may promote prosocial behavior through observer and perpetrator JS.

## Data Availability Statement

The raw data supporting the conclusions of this article will be made available by the authors upon request, without undue reservation.

## Ethics Statement

The studies involving human participants were reviewed and approved by Ethics Committee of the Psychologische Hochschule Berlin. Written informed consent to participate in this study was provided by the participants’ legal guardian/next of kin.

## Author Contributions

RB devised the project and main conceptual ideas. Both authors performed the analysis and wrote the manuscript.

## Conflict of Interest

The authors declare that the research was conducted in the absence of any commercial or financial relationships that could be construed as a potential conflict of interest.
