# Supplementary material for: Justice Sensitivity in Middle Childhood: A Replication and Extension of Findings
Source: Front Psychol. 2021 Jan 25;11:610414. doi: 10.3389/fpsyg.2020.610414 (PMC7868537; doi:10.3389/fpsyg.2020.610414)
Supplement: Supplementary file 1 [file Table_1.pdf]

Table 3

Overview of hypotheses and findings

	hypothesis	previous findings	current study findings	
1	Replication of factor structure	factor structure of correlated, but separable subscales found in middle childhood (Strauss et al., 2020)	three positively correlated, yet distinct subscales for self- and parent-ratings	H1 supported
2	Contrasting patterns of mean-level values in self-and parent-ratings	mean values highest for JS-V and lowest for JS-P when using self-reports, opposite pattern for parent-reports (Strauss et al., 2020)	children reported lowest mean values for JS-V and highest for JS-P, the opposite pattern emerged for parent-ratings	H2 supported
3	negative relations between JS-V and prosocial behavior	negative links between JS-V and prosocial behavior in middle childhood (Strauss et al., 2020)	parent-reported JS-V was negatively related with parent-rated prosocial behavior	H3 supported
4	prediction of prosocial behavior by JS-O and JS-P	parent-rated JS-O and JS-P predicted more prosocial behavior (Strauss et al., 2020)	JS-O and JS-P predicted more prosocial behavior (using parent-ratings)	H4 supported
5	similar correlation patterns with social skills in parent- and teacher-ratings	all JS perspectives positively related to empathy and ToM (using parent-ratings; Strauss et al., 2020)	JS-V, JS-O and JS-P positively related to empathy and ToM; patterns for teacher-ratings similar to but less pronounced than parent-ratings	H5 supported
6	similar re-test reliabilities (compared to previous research)	not yet tested in middle childhood; .43-.46 in previous research in adolescents (Bondü & Elsner, 2015)	re-test reliabilities between r_{tt}=.510 and .593 for parent-reports	H6 supported
7	increases in JS-O and JS-P with age	increases in JS-V and minor changes in JS-O and JS-P during adolescence (Bondü & Elsner, 2015)	significant positive correlation between JS-P/JS-O and age; significant trend towards higher JS-O and JS-P with age (Jonckheere-Terpstra tests using self- and parent-reports)	H7 supported
8	longitudinal associations between JS and prosocial/aggressive behavior	not yet examined in middle childhood	JS-V predicted less prosocial and more aggressive behavior; JS-O and JS-P predicted more prosocial behavior; JS-P predicted less aggressive behavior (only when stability of prosocial and aggressive behavior not considered)	H8 partially supported
9	weak MI between self- and parent-ratings	strict MI between self- and parent-ratings (Strauss et al., 2020)	inspections of fit indices indicated strong MI between self- and parent-ratings	H9 rejected
